# The Effects of Vitamin D Supplementation on Respiratory Infections in Children under 6 Years Old: A Systematic Review

**DOI:** 10.3390/diseases11030104

**Published:** 2023-08-08

**Authors:** Larisa Mihaela Marusca, Gowry Reddy, Mihaela Blaj, Reshmanth Prathipati, Ovidiu Rosca, Felix Bratosin, Iulia Bogdan, Razvan Mihai Horhat, Gabriela-Florentina Tapos, Daniela-Teodora Marti, Monica Susan, Raja Akshay Pingilati, Florin George Horhat, Mavrea Adelina

**Affiliations:** 1 Laboratory Medicine, “Louis Turcanu” Emergency Hospital for Children, Doctor Iosif Nemoianu Street, 300011 Timisoara, Romania; larisa.marusca@umft.ro; 2 Doctoral School, “Victor Babes” University of Medicine and Pharmacy Timisoara, 300041 Timisoara, Romania; felix.bratosin@umft.ro (F.B.); iulia-georgiana.bogdan@umft.ro (I.B.); 3 New York Medical College at St. Mary’s and St. Clare’s Hospital, Denville, NJ 07834, USA; greddy3@primehealthcare.com; 4 Department of Anesthesiology and Intensive Care, Faculty of Medicine, University of Medicine and Pharmacy “Grigore T. Popa”, University Street, 700115 Iasi, Romania; 5 Santiram Medical College General Hospital, Nandyala 518001, India; reshmanthprathipati98@gmail.com; 6 Department of Infectious Diseases, “Victor Babes” University of Medicine and Pharmacy, Eftimie Murgu Square 2, 300041 Timisoara, Romania; ovidiu.rosca@umft.ro; 7 Department of Conservative Dentistry and Endodontics, Faculty of Dental Medicine, “Victor Babes” University of Medicine and Pharmacy Timisoara, Eftimie Murgu Square 2, 300041 Timisoara, Romania; horhat.razvan@umft.ro; 8 Department of Emergency Medicine, Arad County Emergency Clinical Hospital, Calea Victoriei, 310037 Arad, Romania; tapos.gabriela@yahoo.com; 9 Department of Biology and Life Sciences, Faculty of Medicine, “Vasile Goldis” Western University of Arad, Revolutiei Square 94, 310025 Arad, Romania; dana_m73@yahoo.com; 10 Department of Internal Medicine, Discipline of Medical Semiology I, “Victor Babes” University of Medicine and Pharmacy Timisoara, Eftimie Murgu Square 2, 300041 Timisoara, Romania; susan.monica@umft.ro; 11 Malla Reddy Institute of Medical Sciences, Suraram Main Road 138, Hyderabad 500055, India; rjakshay48@gmail.com; 12 Multidisciplinary Research Center on Antimicrobial Resistance (MULTI-REZ), Microbiology Department, “Victor Babes” University of Medicine and Pharmacy, 300041 Timisoara, Romania; horhat.florin@umft.ro; 13 Department of Internal Medicine I, Cardiology Clinic, “Victor Babes” University of Medicine and Pharmacy Timisoara, Eftimie Murgu Square 2, 300041 Timisoara, Romania; mavrea.adelina@umft.ro

**Keywords:** vitamin D, vitamin D deficiency, infection, respiratory infections, children

## Abstract

Childhood respiratory tract infections (RTIs) pose a significant health burden, especially in children under six years old. The main objective of this systematic review was to assess the effectiveness of vitamin D supplementation in the prevention of RTI in this population while also exploring potential effect modifiers such as age, baseline vitamin D status, and type of respiratory infection. A systematic review of the literature published up to February 2023 was conducted according to PRISMA guidelines, searching PubMed, Web of Science, Cochrane, and Scopus databases. Eight studies met the inclusion criteria, which investigated the association between vitamin D supplementation and respiratory infections in children between zero and five years old. The included studies were conducted between 2012 and 2021, encompassing a total of 2189 children from five randomized trials, two case-control studies, and one prospective cohort study. The relationship between vitamin D supplementation and the prevention of childhood RTI was not consistently observed across all included studies. Pooled results demonstrated varied effects of vitamin D supplementation on respiratory infection incidence, severity, and symptoms. Three studies reported statistically significant associations between low vitamin D levels and respiratory infections (OR = 4.90, OR = 6.97), while one study found that children who received vitamin D supplementation of 800 UI/day for 3 months during the cold season had fewer episodes of respiratory symptoms (RR = 0.55) and recovered more quickly from acute RTI. Lastly, according to one study, vitamin D intake < 80 IU/kg/day was significantly associated with the risk of acquiring pneumonia (OR 7.9) but not bronchiolitis. The remaining five studies found no statistically significant differences in infection rates or severity (*p*-value > 0.050). The available evidence on the effectiveness of vitamin D supplementation for preventing and treating respiratory infections in children under six years old is limited, with only a few favorable effects being reported. In some cases, a dose of 80 UI/kg/day was found to provide significant protection for acute respiratory infections, although in the major trials the only benefit was a quicker recovery and fewer respiratory symptoms, with no impact on incidence and severity of respiratory infections. Nevertheless, the study protocol, the supplementation dose, and duration of supplementation had significant variations between studies, leading to inconclusive findings.

## 1. Introduction

Childhood respiratory tract infections (RTIs) are among the most common illnesses encountered in pediatric populations, accounting for a significant proportion of morbidity and mortality in children worldwide [1,2,3]. RTIs during childhood encompass a wide range of infections affecting the upper and lower respiratory tracts, such as the common cold, bronchitis, pneumonia, and bronchiolitis, that are caused by a multitude of viral and bacterial pathogens, with seasonal fluctuations and geographical variations in incidence and etiology [4,5]. Besides the direct impact on children’s health, RTIs place a considerable burden on healthcare systems and families, owing to frequent clinic visits, hospitalizations, and productivity loss [6,7].

Vitamin D is a fat-soluble vitamin that is essential in bone metabolism, immune system regulation, and modulation of inflammatory processes [8]. The synthesis of this essential nutrient occurs in the skin upon exposure to ultraviolet B radiation from sunlight, with dietary intake and supplementation contributing to a lesser extent [9]. Vitamin D deficiency, which is increasingly recognized as a global public health issue, has been linked to numerous acute and chronic diseases, including autoimmune disorders, cardiovascular diseases, and certain types of cancer [10,11,12]. In recent years, the potential role of vitamin D in the prevention and treatment of infectious diseases, particularly respiratory infections, has gained growing interest in the scientific community [13].

A growing body of evidence suggests that vitamin D exerts immunomodulatory and anti-inflammatory effects that may influence the susceptibility to and severity of respiratory infections [14,15]. The proposed mechanisms include the induction of antimicrobial peptides, modulation of T-cell responses, and suppression of excessive inflammatory reactions [16,17,18]. Numerous observational studies have found a link between low levels of 25-hydroxyvitamin D (25(OH)D) in the blood and an increased risk of RTIs. However, when this association has been examined through randomized controlled trials, specifically focusing on the effects of vitamin D supplementation on childhood RTIs, the findings have been inconsistent. Some trials support the idea that additional vitamin D can reduce the risk of RTIs in children, while others do not, indicating that further research is needed to clarify the role of vitamin D in this context.

Given the significant public health implications of RTI and the potential benefits of vitamin D as an affordable, safe, and accessible intervention, it is important to critically appraise and synthesize the available evidence [19,20,21]. A comprehensive understanding of the relationship between vitamin D status and RTI may inform clinical practice and public health policies aimed at reducing the burden of these infections in pediatric populations. Although previous trials and systematic reviews have addressed this topic, with inconclusive results [22,23], there was no focus on a particular type of infection or age range during childhood, when the immune system is developing and is significantly different from adults’ immunity [24,25]. Moreover, respiratory infections are the most common diseases in children, while the preschool age sets a boundary on community exposure to respiratory infections. Thus, the rapidly evolving evidence base necessitates an updated and rigorous evaluation of the literature.

The primary hypothesis of this systematic review is that adequate vitamin D levels are associated with a reduced risk and severity of childhood respiratory infections. Consequently, the main objective was to assess the effectiveness of vitamin D supplementation in decreasing the incidence and severity of RTI in the pediatric population of children between 0 and 5 years old, while the secondary objective was to explore potential effect modifiers, such as age, baseline serum vitamin D levels, and type of respiratory infection. By synthesizing the available evidence, this systematic review aims to elucidate the role of vitamin D in childhood respiratory infections, which may ultimately contribute to the development of targeted interventions and strategies to reduce the burden of these common and impactful illnesses.

## 2. Materials and Methods

### 2.1. Review Protocol

The current study was performed in March 2023, including the following databases: PubMed, Web of Science, Cochrane, and Scopus. The review comprised literature published up to February 2023. The search strategy utilized medical subject headings (MeSH) keywords [26], including the following: “vitamin D”, “vitamin D deficiency”, “25-hydroxyvitamin”, “1,25(OH)2D3”, “25-hydroxycalciferol”, “25-hydroxyergocalciferol”, “children”, “respiratory infections”, “lung diseases”, “upper respiratory tract infection”, “ lower respiratory tract infection”, “common cold”, “human flu”, “influenza”, “pneumonia”, “viral infections”, and “bacterial infections”. Only English-language articles were considered for inclusion.

Following the Preferred Reporting Items for Systematic Reviews and Meta-Analyses (PRISMA) guidelines [27] and the standards set by the International Prospective Register of Systematic Reviews (PROSPERO) [28], we adopted a rigorous, structured search strategy to identify relevant scholarly articles that explore the relationship between vitamin D levels and the incidence of respiratory infections in pediatric populations. This approach ensured methodological integrity and transparency in the search and selection process. In accordance with contemporary scientific practice and to uphold the principles of transparency and replicability, the current study was registered on the Open Science Framework (OSF) platform [29].

The main research question aimed to determine the effects of vitamin D supplementation on the incidence and severity of respiratory infections in infants, toddlers, and preschool children (aged between 0 and 5.9 years). Also, the review examined if there were any effect modifiers and dose-dependent effects of vitamin D supplementation, which would help to identify the optimal dose that could significantly decrease the risk of developing respiratory infections or decrease the severity of RTI in children aged 0–5 years old. The considered effect modifiers were age, baseline vitamin D status of the children, and the type of respiratory infection, that were chosen due to the potential influence they could have on the relationship between vitamin D supplementation and the incidence and severity of respiratory infections in children aged between zero and five years old.

### 2.2. Selection Process

The primary sources of information used in this systematic review consisted of the textual content, tables, figures, and supplementary online resources available within the articles. The selection process began by eliminating duplicate entries, followed by a meticulous assessment of each abstract by two independent researchers to determine its relevance to the research questions. Subsequently, a thorough examination of the complete text was conducted for the remaining articles to ensure that they met the predetermined inclusion criteria. Additionally, two independent researchers performed an in-depth analysis of the reference lists of the collected papers to identify any relevant literature that may have been overlooked during the initial search. This process aimed to enhance the comprehensiveness of the systematic review.

The inclusion criteria for studies in the systematic review were as follows: (1) a study topic on vitamin D supplementation and respiratory infections; (2) serum vitamin D measurement in the studied population of infants (aged 0–1 years), toddlers (aged 1–3 years), and preschool children (aged 3–5 years); (3) the research must have detailed the respiratory infection outcomes; (4) the study cohort must be older than 1 year and younger than 5 years. Conversely, the exclusion criteria were: (1) studies where vitamin D was not measured; (2) studies lacking relevant data on patients’ characteristics and medical history; (3) articles where respiratory infections were not mentioned; (4) studies where the odds ratio (OR), risk ratio (RR), or hazard ratio (HR) was not addressed based on vitamin D supplementation; (5) studies that did not analyze vitamin D supplementation; (6) other exclusions were made for case reports, literature reviews, meta-analyses, letters to editors, and brief communications.

The final analysis was performed encompassing a broad set of variables, including both study characteristics and key findings. In terms of study characteristics, we evaluated: the specific study number and the first author’s name, the geographical location where the study was conducted, the year in which the study was carried out, the research design utilized, and a thorough assessment of the study’s quality. The review encapsulated a comprehensive set of data points such as the total number of patients who participated in each study and analyzed their demographic details, including average or median age and the distribution of genders. Other variables were examined, such as the specific dose of vitamin D administered to the patients, along with the duration of vitamin D supplementation provided. Another crucial aspect that was taken into consideration was the defined threshold for vitamin D insufficiency in each study, alongside the method used for measuring vitamin D levels. Critical insights into the incidence of infections among the patients were gathered, including the type of respiratory infections experienced, their severity, and their association with vitamin D insufficiency as represented by hazard ratios, odds ratios, or risk ratios. Lastly, this review incorporated additional unique factors or features specific to each study, thus ensuring a broad and comprehensive analysis of the collected data.

### 2.3. Data Extraction and Quality Assessment

In this study, we employed the Quality Assessment Tool for Observational Cohort and Cross-Sectional Studies to assess the studies included in our analysis [30]. Each question within the tool was assigned a score of 1 for affirmative responses (“Yes”) and 0 for negative responses (“No”) or other responses, with the aim of determining the final performance score. Studies were classified as fair quality if they scored between 0 and 4, good quality if they scored between 5 and 9, and excellent quality if they scored 10 or higher. To minimize selection bias, missing data, and measurement bias, two researchers independently evaluated the quality of the selected articles. This dual assessment approach enhanced the reliability of the evaluation process. To evaluate the published works, we utilized the Study Quality Assessment Tools provided by the National Heart, Lung, and Blood Institute (NHLBI) [30]. The two investigators individually assessed the studies and documented their findings. These tools were customized to accommodate various study designs, facilitating the identification of methodological or design-related issues.

To assess publication bias, we employed a funnel plot, which involved plotting the standard error of the log odds ratio against its corresponding log odds ratio. The symmetry of the plot was visually examined, and to further evaluate it, we conducted Egger’s regression test. A *p*-value less than 0.05 was considered indicative of significant publication bias. Additionally, we carried out a sensitivity analysis, where we systematically eliminated one study at a time and then recomputed the combined odds ratios. The purpose of this analysis was to evaluate the sturdiness of the outcomes and explore how each individual study might affect the total measure of the effect size.

## 3. Results

### 3.1. Analysis of the Included Studies

**Figure 1:**
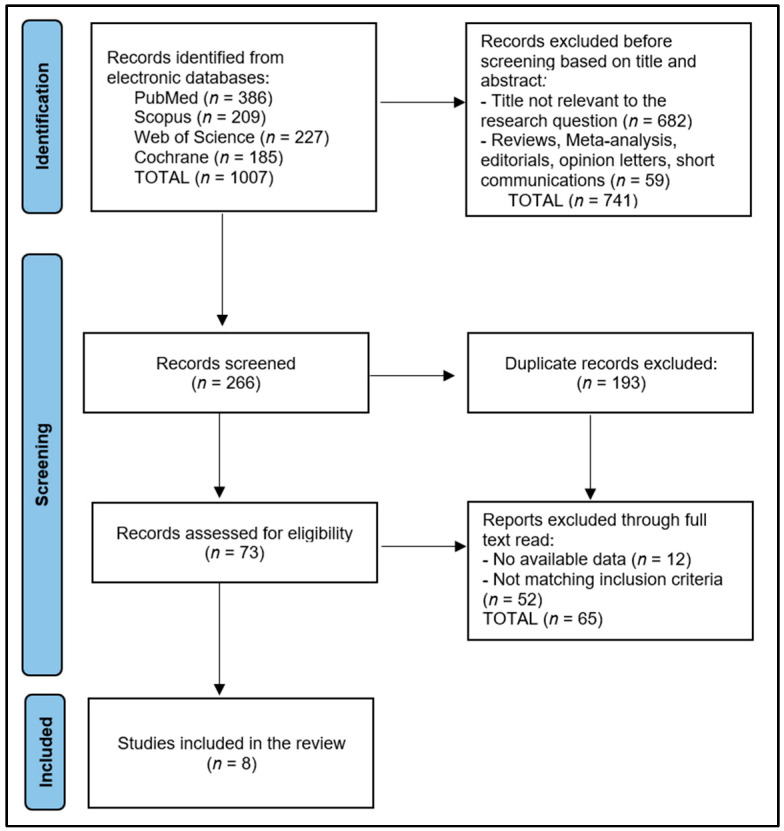
PRISMA Flow Diagram.

In the initial phase of the research process, an exhaustive investigation was undertaken that resulted in the retrieval of a total of 1007 distinct studies. During the subsequent screening procedure, it was identified that 193 of these studies were duplicated, thereby necessitating their exclusion. The preliminary evaluation based on the abstract content resulted in a significant reduction of the study pool. Specifically, 741 studies were eliminated from further consideration due to their lack of alignment with the research objectives (investigating vitamin D supplementation in children under 6 years old), or due to methodological inadequacies that did not specify the study outcome as prevention of respiratory infections. The final stage of the study selection involved a meticulous evaluation of these 73 full-text articles to determine their applicability to the research objectives and their adherence to the predefined methodological criteria. As a result of this rigorous scrutiny, eight studies emerged as meeting all requisite criteria for inclusion in the systematic review, which are referenced in the literature as [23,24,25,26,27,28,29]. This process of study selection and the associated reduction in the number of studies considered for inclusion are illustrated in Figure 1, which provides a clear visual representation of the systematic review’s methodological progression.

Table 1 presents the characteristics of the eight included studies that investigated the effects of vitamin D supplementation on respiratory infections in children under six years old [31,32,33,34,35,36,37,38]. These studies were conducted between 2012 and 2021, with the majority originating from Canada (five studies) [31,32,33,34,36], two from India [35,37], and one from China [38]. In terms of study design, five of these studies utilized a randomized trial approach, while two were case–control studies, and one was a prospective cohort study. Regarding study quality, three studies were rated as excellent [32,33,36], four as good [31,34,35,37], and one as fair [38].

**Figure 2:**
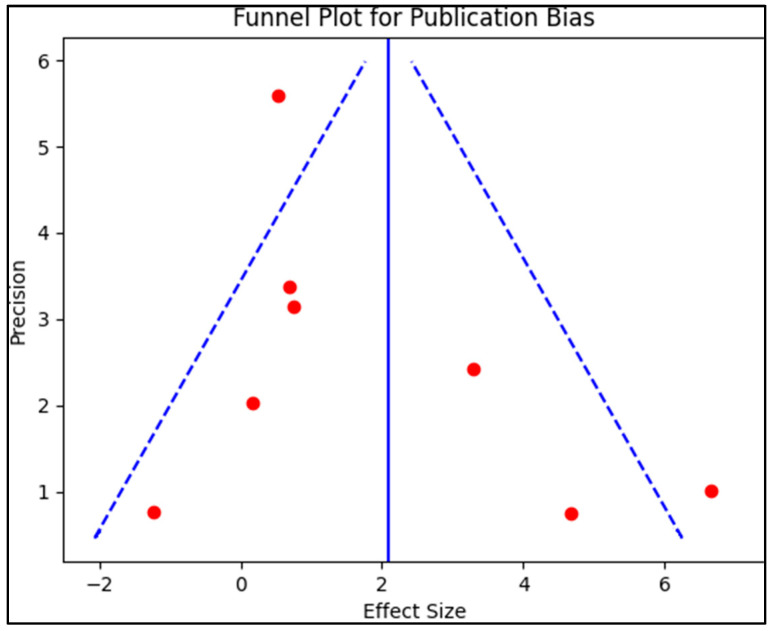
Funnel plot for publication bias.

The majority of randomized trials in this systematic review provided a robust basis for evaluating the potential causal relationship between vitamin D supplementation and the incidence of respiratory infections in children under six years old, considering the consistent results of the bias analysis presented in Figure 2. Randomized trials are considered the gold standard for determining the effectiveness of interventions, as they minimize potential biases and facilitate a more reliable comparison between intervention and control groups. Additionally, the overall good to excellent quality of the included studies further bolstered the credibility of the analysis, ensuring that the conclusions drawn from these studies were based on meticulously conducted research.

### 3.2. Patients’ Characteristics and Interventions

A total of eight studies and 2189 patients were analyzed. The studies demonstrate varying degrees of impact on respiratory infections in children under six years old. In the study by Jensen et al. [32], for example, there was a statistically significant age difference between cases (2.2 years) and controls (3.1 years). In contrast, study 3 [33] and study 4 [34] showed very similar ages for cases and controls, with 2.70 years vs. 2.76 years, respectively. Additionally, the studies by Leis et al., Singh et al., and Jadhav et al. [31,35,37] did not report the average age for cases and controls, as shown in Table 2. These variations suggest that the effect of vitamin D supplementation on respiratory infections in children between 0 and 5 years old may be influenced by factors such as dosage, duration, or other study-specific variables.

### 3.3. Vitamin D Levels and Prevalence of Infections

Table 3 delivers a thorough evaluation of various aspects related to vitamin D, including its levels, insufficiency, the frequency of infections, and the specific infections observed in the studies encompassed by the systematic review. It is important to recognize that the criteria used to define vitamin D insufficiency were not consistent across the studies, with cutoff points ranging from under 20 ng/mL to below 30 ng/mL. This inconsistency in the definition should be considered when juxtaposing outcomes from different studies, as it could influence the perceived connections between vitamin D levels and the likelihood of respiratory infections in children younger than six. Four studies reported on vitamin D levels, invariably displaying higher levels in the case groups as opposed to the corresponding control groups after the intervention. The extent of vitamin D insufficiency was documented in three studies, demonstrating a considerable variation in prevalence rates, from a low of 5.7% to a high of 75.0%. This wide spectrum underscores the variations in the incidence of vitamin D insufficiency across diverse populations and settings.

In the majority of the included studies, the prevalence of infections was lower or comparable among those receiving vitamin D supplementation compared to those who did not take vitamin D supplements, supporting the potential role of vitamin D in reducing the risk of respiratory infections. Furthermore, the types of infections reported in the studies included acute lower respiratory tract (LRT) infections, viral upper respiratory tract (URT) infections, and recurrent pneumonia, as well as a combination of recurrent LRT and URT infections. These different types of infections provide additional context for interpreting the results and suggest that vitamin D supplementation may have varying effects on different types of respiratory infections. The prevalence of infections in the cases and control groups varied across the studies. In general, those receiving vitamin D supplementation demonstrated a lower or comparable prevalence of infections compared to the control group.

### 3.4. Study Outcomes and Vitamin D Effect Measurement

Overall, the research findings concerning the impact of vitamin D supplementation on the severity of respiratory infections and risk evaluation were inconsistent, as detailed in Table 4. Some studies identified substantial reductions in risk, while others found results that were not statistically significant. Specifically, three studies [31,37,38] demonstrated a decreased risk of respiratory infections in the group receiving vitamin D supplementation, with odds ratios (ORs) or relative risks (RRs) ranging from 0.55 to 6.97. Among these, the studies conducted by Leis et al. [31], Jadhav et al. [37], and Xiao et al. [38] produced statistically significant outcomes (with *p*-values of 0.010, *p* < 0.001, and *p* < 0.001, respectively), indicating a meaningful connection between vitamin D supplementation and the risk of respiratory infections.

In contrast, other studies found no statistically significant difference in the risk of respiratory infections between cases and controls. Studies by Jensen et al. [32], Aglipay et al. [33], Hueniken et al. [34], and Singh et al. [35] reported RRs of 0.74, 1.05, 0.97, and 0.69, respectively, but none of these results was statistically significant (all *p* > 0.050). Ducharne et al. [36] also reported a non-significant RR of 0.87 (*p* > 0.050) for asthma exacerbations between cases and controls.

In terms of secondary outcomes or other particularities, several studies reported statistically significant findings. Leis et al. [31] found that vitamin D intake below 80 IU/kg/day was statistically significantly associated with pneumonia (OR 7.9) but not bronchiolitis. Jensen et al. [32] and Hueniken et al. [34] reported statistically significant changes in the mean total 25OHD over the study period but no statistically significant differences in the incidence of infections per patient month of follow-up or total outpatient visits, ED visits, and total antibiotics prescribed. Aglipay et al. [33] found no statistically significant difference in the median time to the first infection between cases and controls (3.29 vs. 3.95 months).

Singh et al. [35] observed that standard therapy with 300,000 IU of vitamin D in children under 6 years old with pneumonia decreased the severity of URT and LRT infections, but the difference was not statistically significant. Ducharne et al. [36] reported no statistically significant change in the mean total 25OHD at 3.5 and 7 months, with hypercalciuria observed in 8.7% vs. 10.3% of cases and controls. Jadhav et al. [37] found that children who received vitamin D supplementation recovered from acute RTI more quickly and had fewer episodes. Finally, Xiao et al. [38] reported a statistically significant reduction in URT and LRT infections after treatment.

## 4. Discussion

### 4.1. Summary and Contributions

The purpose of this systematic review was to explore the impact of vitamin D supplementation on respiratory infections in children younger than 6 years. The results imply that supplementing with vitamin D could have a role in diminishing both the frequency and severity of respiratory infections, though the exact nature of this connection is still ambiguous. Various factors added to the complexity of the findings, such as differences in study methodologies, the characteristics of the participants, and the specific doses of vitamin D supplementation employed across the included studies. Additionally, the evidence is still not definitive, despite observations of enhanced infection outcomes in some studies after vitamin D supplementation, highlighting the need for further research.

The systematic review identified multiple studies [31,32,33,34,35,36,37,38] that reported a significant decrease in respiratory infections among children who received vitamin D supplementation. However, it is important to note that the specific dosage and duration of vitamin D supplementation varied among the studies, making it challenging to establish a conclusive relationship. Moreover, variations in the characteristics of participants, like their initial vitamin D levels and existing risk factors for respiratory infections, could have shaped the results observed. The divergence in outcomes might also be ascribed to disparities in the study populations, geographical locations, and the previously mentioned inconsistency in the definition of vitamin D insufficiency. It is widely recognized that vitamin D levels can differ substantially based on regional influences and skin pigmentation [39,40,41].

Based on the available data, it is challenging to determine the optimal dosing regimen for vitamin D supplementation in children under six years old. A study by Xiao et al. [38] observed a significant reduction in upper and lower respiratory tract infections with 800 IU/day supplementation compared to 400 IU/day. In contrast, Aglipay et al. [33] found no significant difference in the incidence of acute upper respiratory tract infections between children receiving 2000 IU/day and those receiving 400 IU/day. Similarly, the study by Petrovic et al., that was carried out in Europe, showed that participants were generally aware about the meaning and implications of vitamin D hypovitaminosis, but there was no general understanding about the dose and duration of supplementation, since the guidelines are either unclear or contradictory [39]. These discrepancies highlight the need for further investigation to determine the most effective dose and duration of vitamin D supplementation in this population. However, the variations in serum vitamin D levels observed between the study groups might be explained by the differences in the dosages and duration of vitamin D supplementation, as well as possible disparities in baseline vitamin D levels or exposure to sunlight. Most of these studies were conducted in Canada, a country positioned at a high northern latitude where there is reduced sunlight, a factor that could further contribute to these differences.

Our systematic review is in line with some other studies that have investigated the effects of vitamin D supplementation on respiratory infections in children. For instance, a meta-analysis conducted by Martineau et al. [42] showed that vitamin D supplementation reduced the risk of acute respiratory tract infections among all participants, particularly in those with baseline 25-hydroxyvitamin D levels below 10 ng/mL. In our review, studies by Aglipay et al. [33] and Hueniken et al. [34] used a daily dose of 2000 IU, which is higher than the 400 IU used in their respective control groups. Although both studies did not find a significant reduction in the incidence of acute upper respiratory tract infections, their results are consistent with other studies showing a trend towards lower infection rates in the vitamin D-supplemented groups.

The study by Jadhav et al. [37] observed a significant reduction in the risk of recurrent lower and upper respiratory tract infections among children who received vitamin D supplementation. This finding is in line with a study by Yakoob et al. [40], which demonstrated a decrease in recurrent wheezing in children who received vitamin D supplementation. It is also noteworthy that some studies have shown a reduction in the severity of respiratory infections with vitamin D supplementation. For instance, a study by Bergman et al. [43,44] found that vitamin D supplementation was associated with a decrease in the risk of severe bronchiolitis among infants. In our review, Singh et al. [35] reported a reduction in the severity of upper and lower respiratory tract infections with vitamin D supplementation, although the result was not statistically significant.

In four of the studies [31,35,36,37], the research protocol was to administer a bolus of oral vitamin D, followed by low-dose supplementation for a variable amount of time, ranging from 3 months [38] to 12 months [35]. The differences in the duration of supplementation may have also contributed to the inconsistencies in the observed outcomes, as the optimal duration for vitamin D supplementation to prevent respiratory infections in children between zero and five years old remains unclear. Therefore, the discrepancies in the outcomes observed across these studies could be attributed to the differing vitamin D dosages, emphasizing the need for more research to pinpoint the optimal dosage for minimizing the risk of respiratory infections in children under the age of six. Additionally, another factor that may have modified the effects observed is the duration of vitamin D supplementation, which varied significantly among the studies, with ranges from as short as 3 months to as long as 12 months. This variability further underscores the complexity of determining the ideal approach to vitamin D supplementation for this particular health concern. Similarly, the potential effect modifiers that were considered in this study were not established due to the lack of stratification for effect modifiers in the included studies.

The proposed effect modifiers that should be further investigated are age, baseline vitamin D status of the children, and the type of respiratory infection, such as upper or lower respiratory tract infections or bacterial and viral respiratory infections. Age is a significant factor when considering the immune system’s development and its response to infections. For instance, it has been established that infants and younger children have a less developed immune system compared to older children, making them more susceptible to severe infections [45]. Moreover, the absorption and metabolism of vitamin D might also vary with age, further influencing its effectiveness [46]. The baseline vitamin D status could influence the outcomes of vitamin D supplementation. Those who start with a lower vitamin D status might show more marked benefits following supplementation compared to those already having adequate vitamin D levels [47]. Furthermore, the degree of vitamin D deficiency might also dictate the required dosage and duration of supplementation for optimal benefits. Also, the type of respiratory infection could also modify the effect of vitamin D supplementation. Some studies suggest that vitamin D has a more pronounced effect on bacterial infections compared to viral ones [48]. Additionally, the type of infection (lower or upper respiratory tract) could also affect the outcomes due to the variations in immune response.

Despite the general tolerance of vitamin D supplementation, it is worth noting that potential negative side effects may occur if taken in excess, leading to vitamin D toxicity. This condition is associated with symptoms such as hypercalcemia, nausea, vomiting, weakness, and frequent urination [49]. In more severe cases, complications could escalate to include kidney stones, kidney damage, and the calcification of organs or soft tissues. Thus, while vitamin D supplementation can have beneficial effects, there are potential risks that must be carefully considered and managed. Additionally, some studies suggest an association between high vitamin D levels and increased risk of atopic disorders, although the relationship remains controversial and warrants further research [50]. However, vitamin D toxicity remains a rare condition, and the current guidelines do not suggest constant monitoring of 25OHD serum levels in children who supplement their diet with vitamin D, unless a high dose is administered [51].

Based on the study population and geographical position, future research should focus on conducting large-scale, high-quality, randomized controlled trials to determine the optimal dose, duration, and timing of vitamin D supplementation for preventing respiratory infections in children under six years old. Studies should also explore potential differences in the effectiveness of vitamin D supplementation based on factors such as baseline vitamin D status, age, and geographical location. Additionally, researchers should consider evaluating the cost-effectiveness of vitamin D supplementation programs for children between 0 and 5 years old, as well as their potential impact on reducing the burden of respiratory infections in this population.

### 4.2. Strengths and Limitations

Among the limitations of this systematic review are the lack of a rigorous methodology used to optimize search strings, predefined exposures such as the adjustment for the baseline vitamin D levels before starting supplementation, and outcomes such as the type of respiratory infection. Other potential gaps in our methodology are the lack of meta-analysis and TSA analysis and the lack of RoB2 use as a quality assessment tool. Nevertheless, the NHLBI quality assessment tool was used in the current review, and the results of this systematic review provide valuable insight into the potential benefits of vitamin D supplementation in reducing the incidence and severity of respiratory infections among children between 0 and 5 years old. However, there are some limitations considering the heterogeneity of studies in terms of study design, participant characteristics, and vitamin D supplementation doses and durations, making it difficult to draw definitive conclusions. Additionally, some of the included studies had small sample sizes, which may have limited the power to detect statistically significant differences in outcomes. Moreover, the study included too many outcomes to consider like incidence of upper respiratory infections (usually caused by viruses), incidence of pneumonia (usually caused by bacteria), duration of respiratory infection, and severity of respiratory infection which all have different types of modifying factors. This variation makes it challenging to estimate the role of vitamin D supplementation separately and draw specific conclusions. Nevertheless, the preponderance of studies developed in particular parts of the world, such as Canada, may bring a geographical bias, since the obtained data cannot be singlehandedly extrapolated to all races and geographic areas.

## 5. Conclusions

This systematic review provides evidence that vitamin D supplementation does not have a positive impact on reducing the incidence and severity of respiratory infections in infants, toddlers, and preschool children aged between 0 and 5 years old. To some degree, trials show that vitamin D supplementation can benefit this age group with a quicker recovery and fewer respiratory symptoms. However, due to the heterogeneity of the included studies, more research is needed to determine the optimal dose, duration, and timing of supplementation, as significance was not reached in all trials. Nevertheless, a cost–benefit analysis should clarify the need for further trials, considering the previous negative results. In the meantime, regarding the safety of vitamin D supplementation in children under six years old, as described in this study, healthcare professionals should consider the potential benefits of vitamin D supplementation in children at risk for respiratory infections, particularly those with low baseline vitamin D levels or living in regions with limited sunlight exposure.

## Figures and Tables

**Table 1:** Study characteristics.

Study and Author	Country	Study Year	Study Design	Study Quality
1 [31] Leis et al.	Canada	2012	Case–Control	Good
2 [32] Jensen et al.	Canada	2016	Randomized Trial	Excellent
3 [33] Aglipay et al.	Canada	2017	Randomized Trial	Excellent
4 [34] Hueniken et al.	Canada	2019	Randomized Trial	Good
5 [35] Singh et al.	India	2019	Randomized Trial	Good
6 [36] Ducharne et al.	Canada	2019	Randomized Trial	Excellent
7 [37] Jadhav et al.	India	2021	Prospective Cohort	Fair
8 [38] Xiao et al.	China	2021	Case–Control	Fair

**Table 2:** Characteristics of patients and interventions performed in the included studies.

Study Number	Patients (Cases vs. Controls)	Average Age (Years) (Cases vs. Controls)	Gender (Female) (Cases vs. Controls)	Vitamin D Intake (IU)	Duration of Vitamin D Supplementation
1 [31] Leis et al.	197 (105 vs. 92)	NR	40.8% vs. 42.2%	48/kg/day vs. 60/kg/day	6 months
2 [32] Jensen et al.	22 (11 vs. 11)	2.2 vs. 3.1	65.6% vs. 72.7%	1 oral bolus of 50,000 + 400/day	6 months
3 [33] Aglipay et al.	703 (349 vs. 354)	2.70 vs. 2.76	45.2% vs. 39.4%	2000/day vs. 400/day	10 months
4 [34] Hueniken et al.	703 (349 vs. 354)	2.70 vs. 2.76	45.2% vs. 39.4%	2000/day vs. 400/day	4–8 months
5 [35] Singh et al.	100 (50 vs. 50)	NR	44.0% vs. 40.0%	1 oral bolus of 300,000 + 400/day	12 months
6 [36] Ducharne et al.	47 (23 vs. 24)	2.9 vs. 2.9	30.0% vs. 42.0%	2 oral boluses of 100,000 (3.5 months apart)	7 months
7 [37] Jadhav et al.	298 (155 vs. 143)	3.1 vs. 3.2	36.2%	1 oral bolus of 120,000 + 400/day vs. 400/day	6 months
8 [38] Xiao et al.	119 (60 vs. 59)	4.8 vs. 4.8	28.3% vs. 30.5%	800/day vs. 400/day	3 months

NR—not reported; IU—international units; cases—received vitamin D; controls—did not receive vitamin D supplementation.

**Table 3:** Evaluation of Vitamin D levels in children aged 0–5, and the prevalence of respiratory infections.

Study	Vitamin D Insufficiency Threshold	Vitamin D Levels * (Cases vs. Controls) ng/dL	Vitamin D Insufficiency (%)	Incidence of Infections (Cases vs. Controls)	Type of Infection
1 [31]	<20 ng/mL	NR	NR	53.3%	Acute LRT
2 [32]	<30 ng/mL	36.0 vs. 32.0	75.0%	70.8% vs. 76.0	Viral URT
3 [33]	<30 ng/mL	48.7 vs. 36.8	5.7% vs. 22.2%	52.7% vs. 54.5%	Acute URT
4 [34]	<30 ng/mL	48.7 vs. 36.8	NR	52.7% vs. 54.5%	Acute URT
5 [35]	<20 ng/mL	22.9 vs. 19.8	32.6% vs. 55.5%	60.9% vs. 75.5%	Recurrent pneumonia
6 [36]	<30 ng/mL	32.8 vs. 30.5	68.0% vs. 54.0%	87.2%	Acute URT
7 [37]	NR	NR	NR	68.8%	Recurrent LRT and URT
8 [38]	<20 ng/mL	20 vs. 16	NR	NR	Recurrent LRT and URT

* Vitamin D levels after the intervention; NR—not reported; cases—received vitamin D; controls—did not receive vitamin D supplementation; LRT—lower respiratory tract; URT—upper respiratory tract.

**Table 4:** Comparison of respiratory tract infection differences among children aged 0–5 years based on vitamin D serum levels and vitamin D supplementation.

Study Number	Severity of Infection (Cases vs. Controls)	Risk Assessment HR/OR/RR * (95% CI)	Other Study Particularities (Cases vs. Controls)
1 [31] Leis et al.	NR	OR = 4.90 (*p* = 0.010) <80 IU/kg/day (CI: 1.5–16.4)	Vitamin D intake < 80 IU/kg/day was statistically significantly associated with pneumonia (OR 7.9) but not bronchiolitis.
2 [32] Jensen et al.	64.0% vs. 73.0% acute care visits	RR = 0.74 (*p* > 0.050) (CI: 0.46–1.17)	There was a statistically significant change in the mean total 25OHD over the 6-month period, but no statistically significant difference in the URT infections per patient month of follow-up.
3 [33] Aglipay et al.	4.4% vs. 8.5% influenza	OR = 1.05 (*p* > 0.050) (CI: 0.91–1.19)	No statistically significant difference in the median time to the first infection (3.29 vs. 3.95 months).
4 [34] Hueniken et al.	6.6% vs. 6.5% ED visits	RR = 0.97 (*p* > 0.050) (CI: 0.76–1.23)	There was a statistically significant change in the mean total 25OHD at study termination, but no statistically significant decrease in symptoms per winter season, total outpatient visits, total ED visits, or total antibiotics prescribed
5 [35] Singh et al.	19.6% vs. 22.2% severe	RR = 0.69 (*p* > 0.050) (CI: NR)	Standard therapy with 300,000 IU of vitamin D in under-6 children with pneumonia decreased the URT and LRT infection severity, but it was not statistically significant.
6 [36] Ducharne et al.	61 vs. 44 asthma exacerbations	RR = 0.92 (*p* > 0.050) (CI: 0.58–1.49)	There was no statistically significant change in the mean total 25OHD at 3.5 and 7 months. Hypercalciuria was observed in 8.7% vs. 10.3%.
7 [37] Jadhav et al.	7.7% vs. 32.4% (3–4 episodes) after 1 oral bolus of 120,000 + 400/day	OR = 6.97 (*p* < 0.001) <80 IU/kg/day (CI: 3.5–13.8)	Children who received vitamin D supplementation recovered from ARTIs more quickly and had fewer episodes.
8 [38] Xiao et al.	NR	RR = 0.55 (*p* < 0.001) (CI: NR)	Statistically significant reduction in URT and LRT infections after treatment.

* Reference for patients with insufficient vitamin D levels; NR—not reported; cases—received vitamin D; controls—did not receive vitamin D supplementation; IU—international units; URT—upper respiratory tract; LRT—lower respiratory tract; ED—emergency department; CI—confidence interval.

## Data Availability

Not applicable.
